# Resetting Prices: Estimating the Effect of Tuition Reset Policies on Institutional Finances and Enrollment

**DOI:** 10.1007/s11162-022-09723-6

**Published:** 2022-12-20

**Authors:** James Dean Ward, Daniel Corral

**Affiliations:** 1 Chicago, IL USA; 2 Ontario Institute for Studies in Education, University of Toronto, 252 Bloor Street West, Toronto, ON M5S 1V6 USA

**Keywords:** Institutional aid, Enrollment management, Finance, Tuition discounting, Organizational behavior

## Abstract

Private nonprofit colleges are increasingly using tuition resets, or a decrease in sticker price by at least 5%, to attract new students and counter declining demand. While discounting tuition with institutional aid is a common practice to get accepted students to matriculate and to increase affordability, a tuition reset is a more transparent approach that moves colleges away from a high aid/high tuition model. The authors find minimal evidence that these policies increase student enrollment in the long run, but that there may be short-term impacts. As expected, institutional aid decreases and varies directly with the size of the sticker price reduction. The average net price students pay decreases, but this effect may be driven by changes in the estimated non-tuition elements of the total cost of attendance. Finally, net tuition revenue appears unrelated to tuition resets. These findings call into question the efficacy of this practice.

College prices have been increasing at steep rates. The published tuition price at private nonprofit four-year institutions has more than doubled in the past 30 years to an average sticker price of $37,650 in the 2020–2021 academic year (Ma et al., 2020). Although the ultimate price students pay is lower than the sticker price, once subtracting grants, scholarships, and loans, Americans increasingly believe that college is not worth the price (Marken, 2019). The combination of these forces is leading to decreased demand for some private colleges and universities (Krupnick, 2018). For instance, the number of students enrolled across private nonprofit colleges has declined modestly from an all-time high in 2015 (Hussar et al., 2020), and the decline appears to have accelerated during the first two years of the COVID-19 pandemic (National Student Clearinghouse, 2022). These declines are compounded around a growing concern about a potential enrollment cliff due to slowing birth rates and fewer high school graduates in the pipeline to attend college (Grawe, 2018). These declines in enrollment are worrisome as many private colleges are tuition dependent and rely on students to fill seats. Unlike public colleges and universities that have historically relied on state appropriations as their primary revenue source, private colleges rely on student tuition and fees as their main revenue source (Smith, 2019). Private institutions, in particular, are now seeking new enrollment and revenue management strategies to address this declining demand.

In response to decreasing demand, a growing number of private colleges are instituting “tuition reset policies” to attract new students and ostensibly increase demand among students. This practice has increased over time and became an important tool during the pandemic to attract students as demand decreased (Seltzer, 2020). Tuition reset policies simply lower, or reset, the published tuition price by at least five percent in a single given year, though the size of the reset can vary widely (Lapovsky, 2019). Consider the case of Utica College, a small liberal arts college located in the state of New York. In 2016, Utica slashed its sticker price by nearly $14,500 (42%) from the previous year to a published price of $19,996. Utica College is just one of about 63 colleges and universities, and increasing, adopting such a policy across the country in hopes of enhancing enrollment and revenue. This pricing strategy is similar to institutionally funded financial aid, or tuition discounting more generally, which allow institutions to strategically subsidize the cost of attendance for students via grants and scholarships (Breneman et al., 2001; Cheslock, 2006; Hillman, 2012). For example, enrollment managers deploy institutional financial aid as merit-aid or non-need-based aid which allow them to strategically select their incoming class of students by targeting grants toward athletes, economically disadvantaged students, and/or academically successful students (Hillman, 2012). However, tuition resets take an alternative approach to discounting whereby colleges cut published tuition for all potential matriculants instead of allocating aid based on institutional priorities and student needs.

Although deciding which and how many students enroll is important, discounting and reset policies ultimately seek to increase net tuition revenue (i.e., revenue minus aid and other activity costs) (Cheslock, 2006). In a “high aid/high tuition model,” published tuition and fees may be perceived as too high or expensive, however, many students who enroll ultimately benefit from some form of financial aid and thus do not ever pay the advertised cost of attendance (Hearn et al., 1996; Turner, 2018). This high aid/high tuition model allows institutions to benefit from students who would pay this high tuition price, while providing students with limited means or willingness to pay for school an opportunity to enroll by awarding them institutional aid. The high aid/high tuition approach is evidenced by trends over the past two decades in net tuition revenue staying relatively flat among private four-year colleges, despite sticker price increasing at a much higher rate (Ma et al., 2020). This tuition discounting process is more common at private four-year institutions by a factor of nearly two to three times that of public institutions (Baum & Ma, 2010).

Reset policies respond directly to the high aid/high tuition policies many private institutions implement. By lowering the sticker price, families and students may be less intimidated by high college costs and more likely to apply and ultimately enroll in colleges and universities, in theory, leading to increased revenue. However, if net tuition remains steady, as it has over the past 15 years, we expect a decrease in published tuition price to necessitate changes in other net price determinants, namely financial aid expenditures. This study focuses on tuition resets and seeks to examine the policies’ utility as a viable enrollment and revenue management strategy among private bachelor’s-granting colleges. The following research questions guide our study:

1. Does a tuition reset affect enrollment, and do these effects vary over time or by the size of the reset?
2. Does a tuition reset affect institutional aid expenditures, and do these effects vary over time or by the size of the reset?
3. Does a tuition reset affect students’ net price and institutions’ net tuition revenue, and do these effects vary over time or by reset size?

To answer these questions, we use a panel dataset of private nonprofit bachelor’s-granting colleges between 2009 and 2018 and employ a difference-in-differences strategy to estimate effects of these policies. We find that, on average, overall fall enrollment remained relatively stable once institutions adopted these policies. Financial aid expenditures, both total and per full-time-equivalent students, appeared to decrease, as expected, with an overall cut across published tuition price. We also find that net price decreased significantly, however, these decreases were accompanied by decreases in the estimates non-tuition aspects of total cost of attendance. Net tuition revenue per full-time equivalent enrolled student remains stable, suggesting that resets do not yield tuition cost savings for students. These results are robust to several threats to internal validity and design specifications. The results have several implications for institutional policy and practice, chiefly demonstrating that tuition discounts may not be a more effective or sustainable strategy as it relates to enrollment management for revenue maximization over time.

## Literature Review

### Student Price Response

Given the rising cost of college over the last three decades, much research has focused on student enrollment responses to changes in the listed tuition price, known as the Student Price Response Coefficient (Heller, 1997, 1999; Kim, 2010; Langelett et al., 2015; Leslie & Brinkman, 1988; Manski & Wise, 1983). Generally, research on enrollment responses to tuition levels suggests that for every $100 increase in tuition, the probability of enrolling decreases between one and two percentage points, on average, among all students after accounting for economic factors (Heller, 1997, 1999; Kim, 2010; Leslie & Brinkman, 1988). Heller (1999), however, finds that responses vary by significantly by race. For instance, White students are less responsive to price, whereas Black and Hispanic students, across two- and four-year colleges, are much more sensitive to price. Given shifting population dynamics, these differences in price response across demographic groups is important for informing the potential use of tuition resets as a recruitment tool.

Kim (2010) synthesized the literature focusing on the effect of prices on enrollment among private nonprofit institutions. Consistent with the research focused on public college enrollment, they also found negative enrollment responses with tuition increases among private nonprofits. Buss et al. (2004) also find a negative relationship between tuition prices and enrollment at selective liberal arts colleges. The authors provide evidence that both students who do and do not receive financial aid respond negatively to increases in tuition price. Collectively, these studies underscore the inverse relationships between net price and enrollment demands and the role of financial aid.

More recent research has shown negative relationships between price and enrollment in a variety of contexts including online education (Han et al., 2019), at public institutions (Marcotte & Delaney, 2021; Runco et al., 2019), among undocumented students (Conger & Turner, 2015), and among racial and ethnic minoritized students (Allen & Wolniak, 2019). Moreover, qualitative studies have underscored the financial pressures students face such that small increases in tuition and fees can derail students’ educational plans (Goldrick-Rab, 2016; Jones & Andrews, 2021). Taken together, evidence suggests that students respond to changes in prices, thus a decrease in sticker price may impact application and enrollment decisions.

### Review of Tuition Discounting on Enrollment

Institutionally funded financial aid is just one type of financial aid for which students could be eligible, with private scholarships, state aid, and federal aid being the others. On average, research finds that any type of financial aid through the form of grants and scholarships have a positive effect on the probability of enrolling, persisting, and graduating from higher education (Deming & Dynarski, 2010; Nguyen et al., 2019). For example, in a meta-analysis of 43 experimental and quasi-experimental studies estimating the effect of grant aid on persistence and degree completion, Nguyen et al. (2019) found non-repayable grants and scholarships increased both persistence and completion between two and three percentage points. They also found that an additional $1000 in grant aid increased persistence by about two percentage points. This research suggests a positive effect of grant aid across federal, state, and institutional grants and scholarships over and above many of the factors (e.g., proximity, family obligations, etc.) that go into making these series of educational decisions unobserved by colleges. However, few studies have focused exclusively on the provision of institutionally funded financial aid, which is strictly money that the institution provides that are funded by gifts or general funds, and its impact on student enrollment and institutions (Behaunek & Gansemer-Topf, 2019; Hillman, 2012).

Much of the research examining institutionally funded aid has utilized a range of experimental and quasi-experimental techniques to estimate the effect of tuition discounting for students. For instance, Monks (2009) reported on the results of a field experiment where a small private college sent financial aid offers to half of their most highly rated and academically talented students that would otherwise get no financial aid and must pay the published tuition price. The impetus behind this experiment was due to recent budgetary constraints in the number of institutionally funded grants the college could offer. The award offer was a discount of around 17% of the sticker price. Monks found that the experiment was associated with about a three-percentage point increase in the probability of students enrolling.

van der Klaauw (2002) used a regression discontinuity design to estimate the effect of a college’s aid assignment on enrollment decisions from 1989 to 1993. In this analysis, the researcher also estimated the effect of this aid between two different groups: filers (i.e., students who filed and qualified for some type of federal financial aid) and non-filers (i.e., students who did not qualify for financial aid). Using an ability index score based on a combination of academic achievement indicators as the forcing variable, van der Klaauw found that a 10% increase in institutional aid would lead to about an 8.6% increase in the probability of enrolling among filers, those students with the greatest need. Nevertheless, the effect of tuition discounting on non-filers was also positive, albeit to a much smaller magnitude.

Birch and Rosenman (2019) also used a regression discontinuity design, leveraging an eligibility cut off of a combined value representing ACT and GPA, to examine whether an institutional merit aid program affected the likelihood of enrollment among already admitted students. On average, they found that students receiving the scholarship, which was for $2000 a year and renewable conditional on GPA and credit requirements, increased the likelihood of enrolling by 20%. In this setting, the researchers additionally modeled for students that are at the margin of enrolling, controlling for students who are “always certain to enroll” and students “not very likely to enroll.” Birch and Rosenman explain that students within this uncertain group and received the scholarship were around 30% more likely to enroll. The modeling decision of controlling for heterogeneity in the propensity to enroll provided nuance in understanding how tuition discounting can improve the chances of enrolling, particularly for undecided students. Overall, this literature suggests that students are generally sensitive to changes in tuition and specifically increases in institutional aid improves the chances of enrolling.

### Tuition Reset Policies

The existing literature on tuition reset models is thin. Descriptive research offers unclear results regarding the association of these policies to enrollment. Lapovsky (2019) examined these policies at 24 institutions and found average enrollment among first year and transfer students increases two-years after the introduction of these policies at 60% of the institutions. In an analysis of 12 institutions, Armitage (2018) finds that enrollments increase during the year of the reset, but institutions do not necessarily sustain these enrollment gains. This suggests that there may be a temporal component to the effects. A significant limitation of these studies is that they used small samples and descriptive analyses, which are useful approaches to identify previously undocumented patterns and develop hypotheses but cannot provide causal estimates. What is missing from this growing body of literature is the use of quasi-experimental designs to establish plausibly causal relationships between these policies and the outcomes of interest.

## Conceptual Framework

Following the work of Hillman (2012) and Behaunek and Gansemer-Topf (2019), we draw on microeconomic theory of private nonprofit college behavior as our conceptual framework (Breneman, 1994). This theory serves as the underlying framework informing tuition discounting, or institutionally funded financial aid. According to this theory, colleges and universities seek to maximize their utility (e.g., mission, reputation, revenue, etc.) by strategically distributing scarce resources (e.g., financial aid). Maximizing tuition revenue, by increasing enrollment demand, is of particular interest for private colleges given the contemporary economic pressures and circumstances leading them to be even more dependent upon tuition (Breneman et al., 2001; Cheslock, 2006; Hillman, 2012; Price & Sheftall, 2015).

Tuition reset policies specifically seek to increase demand by lowering the sticker price considerably. However, these enrollment changes hinge upon students recognizing the equivalence in net price between a $10,000 scholarship on $30,000 tuition and a flat tuition of $20,000. Discounting practices, especially in the form of prestige-named scholarships, appeal to the emotional side of students by signaling accomplishment and honor (Cheslock & Riggs, 2021). However, there is some risk associated with scholarships as they may depend on academic or athletic performance, participation in specific activities (e.g., orchestra), or fulfilling volunteering hours. From the perspective of a student, a tuition reset reduces that risk by discounting upfront. Prospect theory suggests that when students are aware of the gains of the tuition reset, via communication from the institution, they will prefer this less risky option which will increase demand (Kahneman & Tversky, 2013). From an institutional perspective, a high aid/high tuition model has been used to elicit emotional responses from students and to preserve a sense of quality that is frequently associated with price (Cheslock & Riggs, 2021, Duffy & Goldberg, 2014). However, given that public approval of higher education has decreased over time, much of which is associated with rising costs and the idea that colleges are “greedy” (Bennett, 1987; Marken, 2019), a tuition reset has been seen by institutional leaders as a public commitment to affordability that can work to counterbalance these negative views of postsecondary institutions (Lapovsky, 2019).

While enrollment is on one side of the net revenue coin, net price is on the other. In the case of tuition discounting, when institutional grants increase too much, aid expenditures can outpace sticker prices, thus decreasing net revenue (Behaunek & Gansemer-Topf, 2019; Langelett et al., 2015). Hillman (2012) found in his study of public four-year colleges engaging in institutionally funded financial aid that when tuition discount rates exceed 13%, institutions experience diminishing returns on their investment. A tuition reset moves away from a high aid/high tuition model to a lower aid/lower tuition model. It is unlikely a private institution will fully offsets aid expenditures with a decrease in sticker price. Instead, we hypothesize that a revenue focused institution will decrease aid in a way that maintains or increases net tuition revenue.

## Data

We rely on institution-level data from the Integrated Postsecondary Education Data System (IPEDS) and College Scorecard from 2009 through 2018. We limit our sample to private nonprofit institutions offering a bachelor’s degree. These institutions are among the most expensive undergraduate institutions and the sector most likely to engage in tuition discounting practices (Baum & Ma, 2010). Because sticker prices and discounting practices differ at public institutions and for-profit colleges (Hillman, 2012), our examination of tuition resets focuses solely on private nonprofits. Using IPEDS data, we reduce our sample to all private nonprofit institutions that granted bachelor’s degrees from 2009 through 2018. We further reduced our sample to only include institutions for which all control variables, listed in Table 1, were available for all years of the study. This reduced our sample by 239 institutions. Additionally, we eliminated four additional institutions that reset tuition multiple times from the sample as they likely are unique cases. Table 1 includes descriptive statistics for dropped institutions, where data is available. We do not observe any important deviations from the overall sample, although it may be worth noting the average net price of dropped schools is roughly $6000 less than those in the sample and Hispanic students comprise 13% of students at dropped institutions and 8% at sample institutions.

**Table 1:** Descriptive statistics

	All		Treatment group		Control		Dropped from sample	
	Mean	SD	Mean	SD	Mean	SD	Mean	SD
FTE enrollment	2335	3159	1952	4621	2363	3026	2019	2792
Log FTE enrollment	7.25	1.06	6.78	1.19	7.29	1.04	6.94	1.32
Average net price	23,985	7202	21,813	6180	24,140	7245	18,062	11,421
Log average net price	10.03	0.34	9.95	0.31	10.04	0.35	9.72	0.61
Total institutional aid	3.87E + 07	6.05E + 07	2.71E + 07	5.75E + 07	3.95E + 07	6.06E + 07	2.29E + 07	3.80E + 07
Log total institutional aid	16.76	1.31	16.10	1.51	16.81	1.28	16.07	1.54
Institutional aid per FTE	15,239	7871	13,488	8368	15,364	7819	11,524	12,635
Log institutional aid per FTE	9.51	0.52	9.32	0.65	9.52	0.51	9.14	0.65
net tuition revenue per FTE	18,565	7067	16,027	6349	18,747	7082	17,582	18,490
Log NTR Per FTE	9.76	0.40	9.61	0.38	9.77	0.40	9.64	0.52
Non-tuition COA	13,462	2946	12,965	2496	13,497	2973	12,842	4451
log non-tuition COA	9.48	0.23	9.45	0.21	9.48	0.23	9.39	0.39
Percent American Indian	0.00	0.01	0.01	0.02	0.00	0.01	0.01	0.01
Percent Asian	0.04	0.05	0.04	0.06	0.04	0.05	0.04	0.06
Percent Black	0.12	0.17	0.16	0.19	0.11	0.16	0.09	0.16
Percent Hispanic	0.08	0.09	0.06	0.06	0.08	0.09	0.13	0.27
Percent Haw/Pacific Islander	0.00	0.01	0.00	0.00	0.00	0.01	0.00	0.04
Percent White	0.62	0.21	0.58	0.22	0.62	0.21	0.62	0.28
Percent two or more	0.02	0.02	0.02	0.02	0.02	0.02	0.00	0.01
Percent race unknown	0.07	0.08	0.08	0.09	0.07	0.07	0.07	0.09
Percent non-resident alien	0.05	0.08	0.05	0.12	0.05	0.07	0.04	0.07
Percent pell	0.35	0.16	0.42	0.16	0.34	0.16	0.38	0.26
Admit rate	0.64	0.20	0.66	0.19	0.64	0.20	0.66	0.21
Number of institutions	944		63		881		243	

We define and classify a tuition reset as a reduction of sticker price by five percent or more (Lapovsky, 2019). Because resets are a relatively new strategy and minimal empirical research exists on the effects of this approach, we test various other cut points for a reset in our modeling. Specifically, we use a 10%, 15%, and 20% as alternative definitions of a reset. By testing these alternative definitions, we expect to provide evidence that can support a continued conversation around tuition resets.

**Fig. 1:**
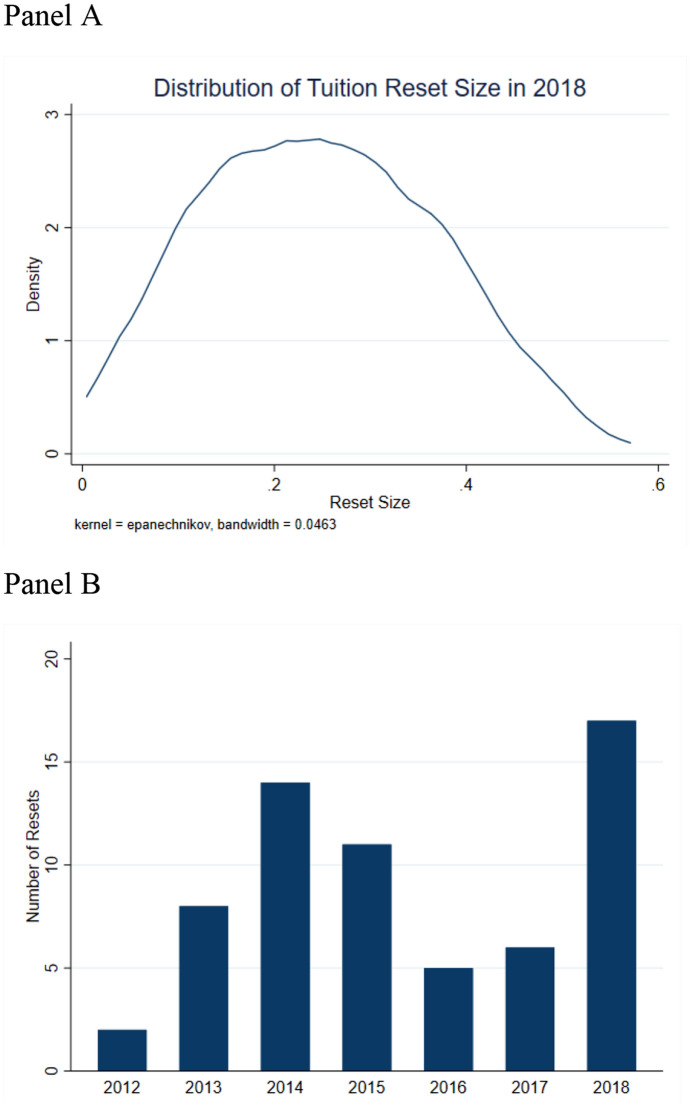
Distribution of reset size and timing

We identify 63 institutions in our sample with a 5% tuition reset from 2012 through 2018. We exclude institutions with a reset prior to 2012 in order to have sufficient pre-treatment data. Panel A of Fig. 1 shows the size of tuition resets in 2018 and Panel B shows the distribution of the number of resets across time. We see that resets vary and are relatively normally distributed between 5 and 53%, and the number of resets are distributed across our study period with peaks in 2014 and 2018. The distribution in the size of the resets will be important for examining the potential for heterogeneous effects across institutions, and also supports our testing of multiple definitions as larger resets may be different than smaller resets.

In this study, we seek to estimate how a tuition reset policy relates to several financial characteristics of an institution. Qualitative research suggests that institutions use resets as a marketing tool to recruit new students and a way to demystify tuition discounting practices and provide more transparency (Lapovsky, 2019). To estimate this relationship, we use six primary outcome variables: full-time equivalent (FTE) fall enrollment, total student aid expenditures, student aid expenditures per FTE, average net price, net tuition revenue per FTE, and non-tuition portions of the cost of attendance (COA). To normalize the data, we take the natural log of each outcome. We examine the effect of a reset on both the net price students pay as well as the non-tuition portion of total COA to understand how students are impacted and how colleges may adjust cost estimates as part of a reset strategy.

In accordance with previous research on tuition prices at private nonprofit college, we include enrollment percentages of IPEDS reported racial and ethnic groups, the proportion of undergraduates receiving a Pell grant, and the average acceptance rate as covariates in our models (Hillman, 2012). A small portion of institution-year observations were missing a reported acceptance rate; for these cases, we imputed an acceptance rate as the average of all other observed years for that institution. Table 1 shows descriptive statistics for the full sample and treatment and control institutions separately. The only substantial difference between the groups we observe is in the proportion of students receiving a Pell grant, which is 42% at reset institutions and 34% at non-reset institutions.

## Methods

To estimate the relationship between tuition resets and our outcomes of interest, we employ a difference-in-differences (DiD) approach. DiD is used to estimate an effect of an exogenous change. We recognize that institutions actively opt into tuition reset policies and thus the policy shift is not truly exogenous. While we do not presuppose our DiD estimates to be causal, we do consider this the best approach to estimate the relationship between tuition reset policies and institutional outcomes.

Our primary estimation strategy is the two-way fixed-effects (TWFE) model:

1 $${Y}_{it}= {\beta }_{0}+{\beta }_{1}{Reset}_{it}+{\gamma }_{i}+{\delta }_{t}+\theta {COV}_{ijt}+{\varepsilon }_{it}$$

where $Y$ is our dependent variable representing our outcomes of interest; *Reset* is an indicator variable equal to one for treated units in the post-policy time period; $\gamma$ and $\delta$ represent institution and year fixed-effects, respectively, that account for unobservable and time-invariant characteristics; and $\theta$ is a corresponding vector of coefficients of additional covariates to improve the internal validity of our model. We cluster our standard errors at the institution level to address serial correlation in outcomes and covariates.

We also test for heterogeneous effects over time and by the size of the reset. In Eq. 2 we interact the treatment indicator with a yearly counter of the length of time since the reset, like an event study design. Colleges can be slow to adapt to changes, and we expect it may take time to balance financial aid expenditures with the tuition reset. Similarly, student demand may vary in relationship to when the reset was implemented.

2 $${Y}_{it}= {\beta }_{0}+{\beta }_{1}{Reset}_{it}+{\beta }_{2}({Reset}_{it}\cdot {\delta }_{t})+{\gamma }_{i}+{\delta }_{t}+\theta {COV}_{ijt}+{\varepsilon }_{it}$$

In Eq. 3, we interact the treatment dummy in the TWFE model with a continuous estimate of the percentage decrease in tuition linked with the reset policy in accordance with recent work by Callaway et al. (2021). This interaction provides an estimate with how the relationship between a tuition reset policy and the outcome of interest varies by the size of the reset. The literature and our conceptual framework suggest that larger cuts in sticker price are likely to induce larger increases in enrollment and larger decreases in financial aid expenditures.

3 $${Y}_{it}= {\beta }_{0}+{\beta }_{1}({Post}_{t}\cdot {Size}_{i})+{\gamma }_{i}+{\delta }_{t}+\theta {COV}_{ijt}+{\varepsilon }_{it}$$

The DiD model relies on an important assumption that the treatment and comparison groups are equal in expectations (also known as the parallel trends assumption). One way to test this assumption is to examine the covariate-adjusted pre-treatment trends in the outcomes. In Eq. 4, we interact a year dummy with a binary indicator, ${Treat}_{it}$, that is equal to 1 for treatment institutions and 0 for control institutions in all years. The coefficient on this provides an estimate if the pre-treatment trend in the outcome of interest varies between the groups, which would invalidate the assumption of the groups being equal in expectation. Because of the staggered implementation, we estimate these parallel trends separately for each cohort of treatment institution. We show these in Appendix Tables 6, 7, 8, 9, and conclude the time trends across the sample are similar enough to meet this assumption.

4 $${Y}_{it}= {\beta }_{0}+{\beta }_{1}{Treat}_{it}+{\beta }_{2}({Treat}_{it}\cdot {\delta }_{t})+{\gamma }_{i}+{\delta }_{t}+\theta {COV}_{ijt}+{\varepsilon }_{it}$$

We also conduct multiple robustness checks on our models. First, we replace our outcomes of interest with outcomes expected to have no relationship with a tuition reset. The purpose of this is to examine the possibility of our main models estimating the effect of some unmeasured exogenous shift that confounds with the tuition reset policy. We use the amount of federal appropriations an institution receives as one alternative outcomes to test our models.

Additionally, previous research suggests the TWFE model may be biased due to the staggered implementation across our treatment institutions (Goodman-Bacon, 2018). The bias may occur because the staggered treatment enables schools to toggle between treatment and control groups. This can result in institutions that reset their tuition in the middle of the treatment period receiving a larger weight in the TWFE estimate of the average treatment effect. To address these concerns, we conduct robustness checks following the work of Callaway and Sant’Anna (2020), Dettmann et al. (2020), Cengiz et al. (2019), and Gardner (2021).

First, we use the *csdid* command in Stata (Rios-Avila, 2021) developed to implement Callaway and Sant’Anna’s estimation strategy for staggered treatments. This approach estimates the average treatment effect by comparing treated units with untreated units thus avoiding comparing treated units across the staggered adoption of tuition resets. Second, we use the *flexpaneldid* command in Stata (Dettmann et al., 2019) to estimate a reweighted average treatment effect. This approach uses matching to identify a single counterfactual for each institution based on the aforementioned covariates and estimates the treatment effect using this smaller sample. Third, we estimate the cohort-specific treatment effects for all schools resetting tuition in each year (Cengiz et al., 2019). This approach provides a series of point estimates that can be examined to determine the potential for an individual cohort to be biasing the average treatment effect identified in TWFE model. Fourth, we use the *did2s* command in Stata (Butts & Gardner, 2021) to employ the two-stage DiD approach to estimating the weighted group-time average treatment effect on the treated institutions.

### Limitations

This study examines private nonprofit colleges and thus the findings are not generalizable to all postsecondary institutions. Additionally, the small sample size of institutions that reset tuition prices may represent a unique type of nonprofit college. Although our models mostly meet parallel trends assumptions, there may be other institutional characteristics that are impacting the effectiveness of tuition reset policies including institution-specific student demand, which may be driven by regional trends, reputation, or marketing materials, among other factors. For example, colleges may accompany a tuition reset with increased marketing activities in order to publicize the change.^1^ We include an institution fixed effect to help account for these institution-specific characteristics, however, more precise measurement of these characteristics would improve our estimation approach. The small sample size may also impact our ability to detect effects with sufficient statistical power. Using Schochet’s (2021) approach for power calculations in a staggered DiD design, we estimate that our minimum detectable effect size with a power of 70% is 0.10.

## Findings

Table 2 provides the primary TWFE estimates from Eq. 1, Table 3 provides the event study estimates from Eq. 2, and Table 4 provides the estimates of heterogeneous effects by reset size presented in Eq. 3. Our robustness checks, discussed below, are included in the appendix in Tables 13, 14, and 15. In Table 5, we present findings from alternate definitions of tuition resets as an additional robustness check and an opportunity to better specify the definition of a tuition reset.

**Table 2:** Main estimates

	FTE enrollment	Total aid	Aid per FTE	Avg. net price	Per FTE net tuition rev	Non-tuition COA
Reset	0.0402	− 0.187***	− 0.227***	− 0.0813***	− 0.0494	− 0.0658***
	(0.0474)	(0.0523)	(0.0398)	(0.0181)	(0.0329)	(0.0190)
Constant	10.85***	21.82***	10.98***	11.17***	9.955***	9.691***
	(1.701)	(2.082)	(0.634)	(0.973)	(0.611)	(0.347)
Controls	Yes	Yes	Yes	Yes	Yes	Yes
Year FE	Yes	Yes	Yes	Yes	Yes	Yes
Institution FE	Yes	Yes	Yes	Yes	Yes	Yes
Observations	9440	9440	9440	9440	9440	9440
R-squared	0.039	0.398	0.440	0.059	0.041	0.164
Number of Institutions	944	944	944	944	944	944

Robust standard errors in parentheses

***p < 0.01, **p < 0.05, *p < 0.1

**Table 3:** Event study estimates

	FTE enrollment	Total aid	Aid per FTE	Avg. net price	Per FTE net tuition rev	Non-tuition COA
Year 1	0.0110	− 0.0358*	− 0.0469*	− 0.00491	− 0.0667*	− 0.0582***
	(0.0207)	(0.0213)	(0.0275)	(0.0157)	(0.0381)	(0.0164)
Year 2	0.0723**	− 0.253***	− 0.325***	− 0.0631***	− 0.0418	− 0.0684***
	(0.0347)	(0.0571)	(0.0527)	(0.0243)	(0.0276)	(0.0222)
Year 3	0.101**	− 0.233***	− 0.334***	− 0.104***	− 0.0287	− 0.0555**
	(0.0471)	(0.0719)	(0.0582)	(0.0314)	(0.0353)	(0.0222)
Year 4	0.127**	− 0.244***	− 0.371***	− 0.0703***	− 0.0508	− 0.0650***
	(0.0572)	(0.0747)	(0.0488)	(0.0257)	(0.0434)	(0.0237)
Year 5	0.0969	− 0.264**	− 0.361***	− 0.118***	− 0.0148	− 0.0856***
	(0.0705)	(0.106)	(0.0645)	(0.0376)	(0.0494)	(0.0273)
Year 6	0.0460	− 0.330*	− 0.376***	− 0.118**	− 0.101	− 0.0729
	(0.142)	(0.200)	(0.0892)	(0.0544)	(0.135)	(0.0506)
Year 7	0.130***	− 0.291***	− 0.421***	− 0.0807	0.0197	− 0.127*
	(0.0498)	(0.0646)	(0.0393)	(0.0808)	(0.0314)	(0.0740)
Constant	0.0110	− 0.0358*	− 0.0469*	− 0.00491	− 0.0667*	− 0.0582***
	(0.0207)	(0.0213)	(0.0275)	(0.0157)	(0.0381)	(0.0164)
Controls	Yes	Yes	Yes	Yes	Yes	Yes
Year FE	Yes	Yes	Yes	Yes	Yes	Yes
Institution FE	Yes	Yes	Yes	Yes	Yes	Yes
Observations	9440	9440	9440	9440	9440	9440
R-squared	0.043	0.404	0.454	0.063	0.042	0.165
Number of institutions	944	944	944	944	944	944

Robust standard errors in parentheses

***p < 0.01, **p < 0.05, *p < 0.1

**Table 4:** Estimated heterogeneous effects by reset size

	FTE enrollment	Total aid	Aid per FTE	Avg. net price	Per FTE net tuition rev	Non-tuition COA
Reset × size	0.220	− 0.763***	− 0.983***	− 0.278***	− 0.194	− 0.280***
	(0.191)	(0.258)	(0.143)	(0.0708)	(0.150)	(0.0824)
Constant	10.86***	21.79***	10.93***	11.16***	9.947***	9.680***
	(1.702)	(2.079)	(0.627)	(0.973)	(0.611)	(0.347)
Controls	Yes	Yes	Yes	Yes	Yes	Yes
Year FE	Yes	Yes	Yes	Yes	Yes	Yes
Institution FE	Yes	Yes	Yes	Yes	Yes	Yes
Observations	9440	9440	9440	9440	9440	9440
R-squared	0.040	0.401	0.447	0.058	0.041	0.165
Number of institutions	944	944	944	944	944	944

Robust standard errors in parentheses

***p < 0.01, **p < 0.05, *p < 0.1

**Table 5:** TWFE using alternate tuition reset definitions

	FTE enrollment	Total aid	Aid per FTE	Avg. net price	Per FTE net tuition rev	Non-tuition COA
10 Percent	0.0518	− 0.208***	− 0.260***	− 0.0867***	− 0.0385	− 0.0777***
	(0.0542)	(0.0591)	(0.0432)	(0.0201)	(0.0357)	(0.0213)
15 Percent	0.0714	− 0.212***	− 0.283***	− 0.0852***	− 0.0589	− 0.0756***
	(0.0552)	(0.0640)	(0.0442)	(0.0219)	(0.0367)	(0.0232)
20 Percent	0.0846	− 0.247***	− 0.332***	− 0.0873***	− 0.0360	− 0.0877***
	(0.0600)	(0.0766)	(0.0434)	(0.0226)	(0.0447)	(0.0279)

### Main Estimates

Our first research question asks whether a tuition reset policy is associated with changes in enrollment, and whether these effects vary over time or by size of the reset. While we do not find evidence of a significant average treatment effect across all treatment years, as shown in Table 2, Table 3 provides evidence there may be a short-term increase in enrollment. Point estimates suggest a 7–13% increase in FTE undergraduate enrollment in years two, three, and four following the reset. However, the point estimate for year one of the reset is 0.01 and statistically insignificant. We discuss this pattern in more detail below in our analysis of the staggered timing effects. The estimate in Table 4 suggests that reset size is unrelated to enrollment changes following the change.

Our second research question seeks to understand the relationship between tuition resets and financial aid expenditures. We examine both the change in total financial aid and the per FTE change. We find an average decrease in total institutional aid dollars of 19%, and a decrease of 23% for per FTE aid following the reset. The decrease in both total aid and aid per FTE appears to remain relatively stable with some change in magnitude over time (i.e., larger decreases in aid), as seen in Table 3. Larger tuition resets are also associated with larger cuts to total and per FTE aid expenditures. Because the size of the reset is measured as a percentage from 0 to 1, the coefficients in Table 4 should be interpreted such that a 10% cut in tuition is associated with a 7.7% reduction in total student aid and a 9.9% reduction in per FTE aid. This interpretation assumes a linear relationship between the size of the reset and changes in student aid expenditures.^2^ Under this assumption, there appears to be nearly a one-to-one trade off in the percentage changes of tuition resets and per FTE aid expenditure, whereas total aid budgets appear to decrease at a slower rate as the size of the reset increases.

Our third research question concerns the combination of expected changes in enrollment with expected changes in student aid expenditures following a tuition reset. We seek to estimate how the average net price for undergraduates and net tuition revenue for institutions relate to tuition reset policies. We find evidence that the average net price decreases by roughly eight percent following a tuition reset. This effect seems to grow over time with point estimates in later years suggesting net price decreases by 12%. We also find that larger tuition resets result in larger reductions in net price. Using the approximate tails of the distribution of reset sizes in our study, a five percent reset is associated with a 1.4% decrease in net price and a 50% cut in sticker price is associated with 14% decrease in net price, assuming a linear relationship between effect and reset sizes. However, decreases in net prices for students are also accompanied by decreases in the non-tuition portion of a student’s total cost of attendance suggesting that the “savings” students experience may be attributable to the college estimating lower cost of living expenses (e.g., housing, food, transportation, etc.) rather than a decrease in the net tuition price students pay. The magnitudes of these decreases in non-tuition COA expenses closely resemble those of the net price across Tables 2, 3, 4.

We also examined the effect of a tuition reset on net tuition revenue for institutions. The point estimate in Table 2 indicates a 5% decrease in net tuition revenue per FTE, however it is accompanied by a large standard error and is statistically insignificant. Moreover, the event study estimates show significant variability in estimates over time and few are statistically significant; there also does not appear to be a relationship between net tuition revenue and the size of the reset, as seen in Table 4. Not only does this suggest that tuition resets are not an effective way to increase per FTE net tuition revenue, it provides additional support for the idea that students’ reduced net price is largely driven by non-tuition factors.

### Falsification and Robustness Checks

As a falsification check, we estimate these models using an alternative outcome that is not expected to be related to a college implementing a tuition reset: total federal appropriations. We find no evidence of effects on unrelated outcomes, which provides additional support that the effects on our primary outcomes of interest are related to the tuition reset policy. We provide results of this falsification test in Table 12 in the appendix.

Table 13, in the appendix, provides the Callaway and Sant’Anna estimates of the average treatment effects. These estimates closely match the TWFE estimates provided in Table 2 which suggests, in this specific case, the differential timing has minimal effects on the TWFE estimator. We also address the potential bias that exists due to the staggered implementation by providing estimates of the average treatment effect in accordance with the estimation strategy put forward by Dettmann et al. (2019). We present these estimates in Table 14, in the appendix. Because this approach uses matched pairs, the sample size is small and thus the standard errors are large. The estimates from this approach, however, generally confirm the size and direction of our estimated effects. The coefficients suggest a modest increase in enrollment and large decreases in student aid. While the DiD estimate for enrollment is similar to the TWFE model, the mean differences in treatment and matched control institutions suggest the effect is possibly driven by both an increase in enrollment at reset schools and a decrease at non-reset schools. Because these are matched pairs, this finding points to the possibility of students sorting across similar schools based on reset policies. The modest decrease in net price is accompanied by a small decrease in non-tuition COA. The effect on net tuition revenue is near zero, which is accompanied by virtually no change in tuition revenue from treatment or matched control institutions. These similarities between the TWFE, Callaway and Sant’Anna, and flexible conditional ATE coefficients are important for the validity of our estimates of the heterogeneous treatment effects presented in Table 4 as the approach we take in Eq. 3 does not account for the staggered timing and solely isolates the heterogeneity associated with the continuous measure of reset size.

**Fig. 2:**
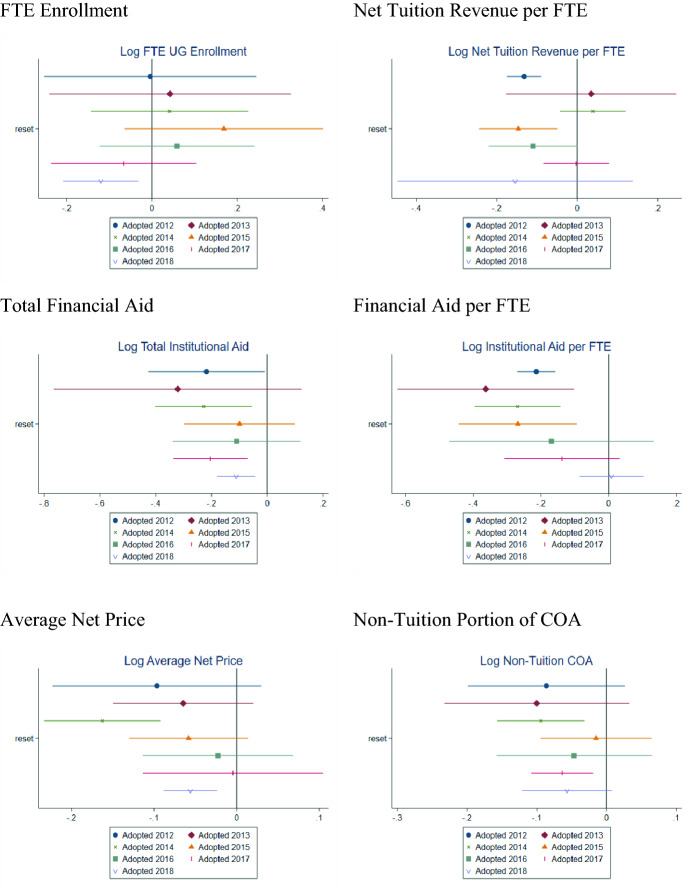
Cohort-specific estimates

To further examine potential sources of bias in the TWFE estimates, we estimate cohort-specific effects following the approach of Cengiz et al. (2019). Because our treated sample only includes 63 institutions, each yearly cohort of treated institutions is small and thus the standard errors for the cohort-specific estimates are large. However, the point estimates can help identify patterns across cohorts and potential outliers that bias the TWFE estimates. Figure 2, in the appendix, provides the cohort-specific estimates for each of the four outcomes of interest.

The mix of positive and negative estimated effects aligns with the lack of specificity in our main estimate of the relationship between tuition resets and enrollment. The cohort-specific estimates show some evidence that late adopters may see decreases in enrollment despite the fact that our time-trend estimates in Table 3 suggest positive enrollment effects in the early years following adoption (late adopters would only have a small number of treatment years and thus would only be in the early year event study estimates). It is important to also consider that while the parallel trends assumption is generally met, it does not hold well for the 2017 cohort of adopters, which puts into question the validity of that specific estimate. This is important given that the preceding years are generally positive, albeit statistically insignificant. These points, coupled with the large standard error on the TWFE estimate of the enrollment change, lead us to be confident in our main finding that there does not appear to be a strong long-term enrollment effect from the tuition reset, but there may be short-term increases.

Decreases in total aid appear to be consistent across cohorts, but per FTE aid decreases are concentrated among the earlier adopters. Decreases in the average net price are relatively consistent, although the earlier adopters may decrease their net price more than the later adopters. The 2013 cohort shows evidence that it fails the parallel trends assumption and also has the most extreme cohort-specific estimated effect. If we exclude this specific estimate, we still identify a negative relationship, but one that more closely mirrors the magnitude of effects on the non-tuition COA, thus supporting the idea that colleges may decrease net price through non-tuition levers. Additionally, the clustering among earlier cohorts may help explain the larger effect we find in our event study in the later years, which would consist solely of the initial institutions that reset tuition.

The parallel trends for the per FTE net tuition revenue show some weaknesses for the 2013 and 2017 cohort. The cohort-specific estimates for these two groups are also roughly 0, compared to mostly negative estimates for other groups. While these two cohorts may play a role in upwardly biasing the TWFE estimate, or moderating the negative effect, we are still confident in our main finding that resets do not appear to be related to net tuition revenue given how large the standard errors are in our various estimation strategies.

In Table 15, in the appendix, we estimate the two-stage DiD ATE which aligns closely with our other estimates thus providing additional evidence of our primary set of findings. Gardner’s (2021) approach uses a weighting scheme that results in the years soon after a policy change influencing the estimated ATE more than years further in the future (Cunningham, 2021). Given that tuition resets can be erased over time by tuition increases, especially smaller resets, and that the reset is usually accompanied by some marketing and publicity efforts that may have a stronger impact in the near term, we believe the two-stage DiD is a strong confirmation of our TWFE models. Despite the higher weight associated with short-term outcomes, the ATE for enrollment is still statistically insignificant.

Finally, we examine alternative definitions of a tuition reset. While Eq. 3, and the findings presented in Table 4, show a negative relationship between reset size and financial aid expenditure, net price, and non-tuition COA, using differing cut points for the reset definition allows us to compare the magnitude of estimates associated with larger resets. The findings in Table 5 suggest stronger effects on financial aid expenditures, with larger decreases in aid occurring at institutions that cut tuition by larger amounts. The net price and non-tuition COA are more stable across definitions, but the magnitude grows marginally with larger resets. These alternative definitions also help account for potential decreases in tuition that were not intentional or well publicized resets or were the result of an institution’s change in IPEDS reporting process (e.g., from flat tuition to per credit hour tuition rate).

To summarize, the falsification and robustness checks largely confirm our primary estimates that tuition resets appear to have minimal effects on enrollment in the long-run, to result in decreases in total and per FTE institutional aid, to be associated with lower average net prices and non-tuition COA estimates, and to have little impact on net tuition revenue per FTE. The main estimates do suggest that there may be a positive effect on enrollment in the first few years following the reset, but that the effect dissipates over time. We also show evidence that spending on institutional aid, the net price, and non-tuition COA decrease as the tuition reset increases.

## Discussion

### Enrollment

We do not find strong evidence that tuition reset policies are closely related to increases in long-term enrollment, although there is some evidence of a near-term growth. These effects are important considering the declining number of “traditional-aged” students (Grawe, 2018), who are the typical student at private nonprofit colleges, and the declining faith and interest in higher education (Marken, 2019). Tuition-dependent colleges will need to maintain enrollments for their continued operations, thus better understanding how students respond to resets is important. A range of factors related to the college-going decision may be driving these findings. First, price may not be the most determinative factor for students, or resets may not be significant enough to counteract sticker shock (Levine et al., 2022). That is, despite the reduction in sticker price, students may still opt to enroll elsewhere for non-financial reasons. While prospect theory suggests that the security associated with a tuition reset or a guaranteed discount (Kahneman & Tversky, 2013; Tversky & Kahneman, 1985)—as opposed to a discount through institutional aid that may be contingent on continued performance—would be an important incentive for students to enroll, our findings do not provide evidence that students are responding this way, except potentially in the short run. Although one explanation is that resets are too small, even using a more restrictive cutoff of 20% as the definition of a reset does not produce evidence that enrollments respond. However, if the reset is not maintained over time, the security associated with the lower tuition likely fades over time which may be contributing to the short-lived effects. Other scholars note the emotional and psychological draw that discounting through prestige-granting scholarships has for students (Birch and Rosenman, 2019; Cheslock & Riggs, 2021; Monks, 2009; van der Klaauw, 2002), which may subvert the potential enrollment effects of a tuition reset, especially if the decreased institutional aid came from cuts to these types of scholarships. A direct comparison of these theories within the context of tuition resets would provide important insights into student decision-making.

Second, students may perceive the reset as an indication of poor quality. Private institutions, which hold the highest sticker prices, may be a Veblen good; that is, a higher price may indicate a higher value to consumers (Veblen, 2005). In the past, institutions have used this Chivas Regal effect to bolster status. For example, when institutions fall in national rankings, they respond by increasing tuition prices in an attempt to increase perceived quality (Askin & Bothner, 2016). However, we posited that colleges may be lowering sticker prices to counter public narratives and outcry about the high price of college and to publicly show a commitment to affordability in an effort to become a more attractive option for students. Additional research into the effects of sustained resets would help understand these enrollment effects. Analyses using student-level qualitative and quantitative data should be considered to better understand these dynamics.

Third, despite the reset, parents and students may continue to make errors in predicting cost and ultimately overestimate how expensive the institution will be. These errors may be minimized during the initial decrease in tuition, but erased as recency of the reset fades. This factor is particularly salient for low-income and racially minoritized students (Grodsky & Jones, 2007), and suggests an avenue for further consideration.

Fourth, early effects on enrollment suggest a tuition reset may be an effective marketing tool in the short term. However, as sticker prices increase following the reset, that marketing ploy is unlikely to help. It is worth considering that long-term effects may not even be the goal or expectation given the general upward trajectory of tuition in the long run. Like other marketing tools, administrators may only expect a short-term effect. Additionally, the flexible conditional ATE presented in Table 14, while statistically insignificant, suggest that any enrollment effect may be the product of institutions with a reset experiencing small gains in enrollment while matched institutions see declines in enrollment. It is possible that near-term enrollment gains after a reset reflect a siphoning of students from similar institutions. Moreover, the short-term enrollment effects we see suggest that a tuition reset policy is not likely to be a viable solution to any long-term downward trends in student demand. Instead, the reset may only be a temporary fix to flagging enrollments. These findings strengthen the call for continued research into students’ college-going decisions.

### Institutional Aid

At private nonprofit colleges, discounting the sticker price is a longstanding and increasingly common practice (Ma et al., 2020). It is not surprising that sticker prices and discount amounts have increased jointly. For private institutions, the average net price students pay dictates the total tuition revenue and thus a significant portion of most private nonprofit colleges’ budget (Archibald & Feldman, 2014). As we describe above, a tuition reset is logically accompanied by a decrease in institutional aid. Our findings support this. Both the total amount of aid and per FTE aid decrease following a reset. Moreover, the decreases in aid vary directly with reset size. That is, when a school cuts tuition by a greater percentage, it decreases institutional aid by a larger percentage compared to smaller tuition resets. This finding confirms the importance of net tuition revenue for colleges and that institutions use dynamic pricing that combines sticker price with aid awards.

Behaunek and Gansemer-Topf (2019) examined the relationship between tuition discounting and enrollment outcomes—student applications, enrollment numbers, admission rate, and enrollment by race and income—at small private baccalaureate colleges. Using a ten-year panel data set of private colleges, they found the number of institutions providing more than 95% of their incoming first-year students financial aid has increased from 2003 to 2012. During this time, there have been steady increases in the number of racial/ethnic minoritized students and Pell recipients enrolling in these colleges, while admission rates are down. From an equity perspective, it is important that future research examine the relationship between tuition reset policies and changes in the composition of student enrollments, and how these changes compare to other discounting strategies. Reset policies have the potential to be a tool to lower the perceived cost barrier for historically underserved students and thus increase access.

### Net Price and Tuition Revenue

The combination of the decrease in sticker price and the decrease in institutional aid associated with a tuition reset policy combine to have an effect on the average net price students pay. Our findings show a negative impact on net price. Although colleges that reset tuition also decreased financial aid, this decrease does not appear to be enough to fully offset the drop in sticker price. This means colleges that reset tuition are, on average, less expensive than they previously were. However, the decrease in net price comes with a decrease in non-tuition COA. The price students pay include these non-tuition elements such as books, supplies, room, board, and transportation. Following a reset, institutions lower the expected cost of these expenses by five to eight percent. A tuition reset, however, is only a reduction of the published tuition price, not a change in the costs of transportation or books. We know that colleges typically accompany their reset with substantial marketing and publicity efforts (Lapovsky, 2019), and our findings suggest they may employ strategies that make the overall cost of attendance appear lower by reducing the estimated cost of non-tuition expenses. While this may be an additional marketing or recruitment tool, it may also reflect an effort to recalibrate estimated costs alongside the tuition reset. Future research should examine the accuracy of these changes to non-tuition COA to better discern between an earnest recalibration and marketing tool.

### Net Tuition Revenue

This decrease in net price runs counter to larger trends among private nonprofit colleges. Despite increasing levels of discounting, colleges have maintained a relatively flat net price (Ma et al., 2020). Our findings suggest that per FTE net tuition revenue remains stable after a reset. While point estimates are negative, they are statistically insignificant across our models and robustness checks. Although some colleges may use a reset for marketing purposes or as a public commitment to affordability, they adjust aid expenditures in a way that leaves per FTE net tuition revenue flat. In other words, students are still giving institutions the same amount of tuition before and after a reset.

Private nonprofit colleges have largely embraced the high aid/high tuition model, but a tuition reset may indicate a shift to a low aid/low tuition model where sticker price is closer to the actual net price paid. A tuition reset essentially redistributes institutional discounting evenly across students, rather than having enrollment managers award dollars to individual students on an ad hoc basis. Because merit-aid, which comprises a growing share of institutional discounts, is awarded disproportionately to wealthy and white students, a tuition reset may create a more egalitarian set of net prices across groups of students. It is important for future research to examine the potential equity impacts of such a shift away from the high aid/high tuition model, even if the average net tuition revenue remains flat.

## Conclusion

In this study we examine the relationship between tuition reset policies and enrollment, institutional aid expenditures, and net tuition prices. Our findings suggest that colleges behave in a revenue maximizing manner and reduce institutional aid as they cut sticker prices. We provide evidence that institutions with the largest tuition resets also cut aid by the largest amounts. Although colleges may be revenue maximizing in their strategies, tuition resets do not appear to have significant or sustained effects on enrollment and are unlikely to help counter flagging demand. Counter to expectations, net price decreases following a reset. However, this decrease is accompanied by a decrease in non-tuition COA which suggests colleges may reduce the expected costs of books, transportation, and other non-tuition expenses as an earnest recalibration of costs during the reset process or as an additional way to market lower prices to students. Importantly, net tuition revenue per FTE does not appear to decrease after a tuition reset, suggesting students are still facing similar tuition bills. We believe it is important for future research to discern between these approaches to better understand reset policies.

This study is among the first to explore the effects of a tuition reset policy. Although we find minimal overall effects on enrollment, additional research on the promise of tuition reset policies to increase access for underserved groups of students is important. Additionally, we suggest future work examine student decision making in the face of a reset. While we used a five percent cutoff as our definition of a tuition reset to mirror previous research (Lapovsky, 2019), we explored how the effects vary with the size of the reset and what the effects were using alternative cutoffs. The effects were consistent in their significance across these tests and the magnitude appeared to grow as the reset grew. The variability in reset sizes and effects leads us to conclude that more attention should be paid to defining resets. Scholars examining this topic should also consider differentiating resets by other accompanying factors, such as change non-tuition COA simultaneously. Bringing additional nuance to the definition of a tuition reset can help future work more clearly identify effects and the contexts in which they occur.

## Appendix

See Tables 6, 7, 8, 9, 10, 11, 12, 13, 14, 15 and Fig. 2.

**Table 6:** Parallel trends FTE enrollment, by cohort

	2012	2013	2014	2015	2016	2017	2018
Treat	0.0962	− 0.0168	− 0.00944	− 0.196	− 0.00369	0.144	0.163***
	(0.184)	(0.164)	(0.133)	(0.173)	(0.132)	(0.0998)	(0.0606)
2010	− 0.0870	− 0.0209	− 0.0364	− 0.0637	− 0.0334	− 0.0201	− 0.00936
	(0.0667)	(0.0424)	(0.0350)	(0.107)	(0.0350)	(0.0123)	(0.0147)
2011	− 0.189*	− 0.0209	− 0.0107	0.0174	− 0.0781**	− 0.0480***	0.00478
	(0.111)	(0.0407)	(0.0558)	(0.102)	(0.0343)	(0.0177)	(0.0265)
2012		− 0.0596	− 0.0496	0.0129	− 0.0195	− 0.0729***	− 0.00294
		(0.0785)	(0.0567)	(0.140)	(0.0315)	(0.0243)	(0.0449)
2013			− 0.0598	0.0713	− 0.0607	− 0.0916***	− 0.0117
			(0.0793)	(0.155)	(0.0526)	(0.0314)	(0.0456)
2014				0.126	− 0.0955	− 0.133***	− 0.0740
				(0.179)	(0.0797)	(0.0380)	(0.0718)
2015					− 0.0904	− 0.117**	− 0.0440
					(0.114)	(0.0557)	(0.0410)
2016						− 0.141*	− 0.111
						(0.0845)	(0.0714)
2017							− 0.136**
							(0.0557)
Constant	10.96***	10.91***	11.07***	10.94***	10.87***	10.98***	10.90***
	(1.724)	(1.707)	(1.727)	(1.735)	(1.706)	(1.727)	(1.720)
Institution FE	Yes	Yes	Yes	Yes	Yes	Yes	Yes
Year FE	Yes	Yes	Yes	Yes	Yes	Yes	Yes
Controls	Yes	Yes	Yes	Yes	Yes	Yes	Yes
Observations	8830	8890	8950	8920	8860	8870	8980
R-squared	0.042	0.042	0.043	0.047	0.042	0.043	0.045
Number of institutions	883	889	895	892	886	887	898

Robust standard errors in parentheses

***p < 0.01, **p < 0.05, *p < 0.1

**Table 7:** Parallel trends total institutional aid, by Cohort

	2012	2013	2014	2015	2016	2017	2018
Treat	0.294**	0.307	0.174	0.125	0.111	0.194*	0.152***
	(0.143)	(0.216)	(0.129)	(0.169)	(0.172)	(0.112)	(0.0507)
2010	− 0.100**	0.0301	0.0266	− 0.0846	− 0.0519	0.0409	− 0.0371
	(0.0404)	(0.0493)	(0.0404)	(0.164)	(0.0816)	(0.0546)	(0.0226)
2011	− 0.129*	0.0418	0.0796	− 0.0447	− 0.117	0.0468	− 0.0163
	(0.0744)	(0.0526)	(0.0555)	(0.130)	(0.0733)	(0.0781)	(0.0280)
2012		− 0.0165	0.0651	− 0.0115	0.0941	0.0191	− 0.0280
		(0.0609)	(0.0783)	(0.156)	(0.142)	(0.0812)	(0.0347)
2013			0.100	− 0.0113	0.0103	0.0259	− 0.0138
			(0.101)	(0.166)	(0.0999)	(0.0797)	(0.0467)
2014				0.00168	− 0.0113	− 0.0140	− 0.0395
				(0.178)	(0.118)	(0.0945)	(0.0402)
2015					0.0688	− 0.0146	− 0.00516
					(0.219)	(0.0972)	(0.0499)
2016						− 0.0277	− 0.0877
						(0.0843)	(0.0635)
2017							− 0.133**
							(0.0632)
Constant	21.53***	21.48***	21.64***	21.56***	21.69***	21.55***	21.49***
	(2.121)	(2.111)	(2.121)	(2.138)	(2.079)	(2.123)	(2.112)
Institution FE	Yes	Yes	Yes	Yes	Yes	Yes	Yes
Year FE	Yes	Yes	Yes	Yes	Yes	Yes	Yes
Controls	Yes	Yes	Yes	Yes	Yes	Yes	Yes
Observations	8830	8890	8950	8920	8860	8870	8980
R-squared	0.432	0.418	0.425	0.425	0.431	0.432	0.432
Number of institutions	883	889	895	892	886	887	898

Robust standard errors in parentheses

***p < 0.01, **p < 0.05, *p < 0.1

**Table 8:** Parallel trends institutional aid per FTE, by Cohort

	2012	2013	2014	2015	2016	2017	2018
Treat	0.198***	0.324**	0.184*	0.321**	0.115	0.0502	− 0.0111
	(0.0464)	(0.128)	(0.108)	(0.136)	(0.180)	(0.150)	(0.0547)
2010	− 0.0133	0.0510**	0.0630	− 0.0209	− 0.0185	0.0610	− 0.0277
	(0.0275)	(0.0249)	(0.0423)	(0.0832)	(0.0531)	(0.0538)	(0.0208)
2011	0.0600	0.0627*	0.0904*	− 0.0621	− 0.0390	0.0948	− 0.0211
	(0.0397)	(0.0334)	(0.0545)	(0.0726)	(0.0619)	(0.0769)	(0.0264)
2012		0.0431	0.115*	− 0.0245	0.114	0.0920	− 0.0251
		(0.0677)	(0.0638)	(0.0864)	(0.119)	(0.0729)	(0.0347)
2013			0.160	− 0.0826	0.0710	0.118	− 0.00211
			(0.119)	(0.100)	(0.0686)	(0.0767)	(0.0251)
2014				− 0.124	0.0842	0.119	0.0345
				(0.150)	(0.0848)	(0.0868)	(0.0607)
2015					0.159	0.103	0.0388
					(0.154)	(0.118)	(0.0330)
2016						0.113	0.0230
						(0.139)	(0.0354)
2017							0.00332
							(0.0414)
Constant	10.57***	10.57***	10.57***	10.62***	10.82***	10.57***	10.59***
	(0.628)	(0.633)	(0.630)	(0.634)	(0.616)	(0.627)	(0.622)
Institution FE	Yes	Yes	Yes	Yes	Yes	Yes	Yes
Year FE	Yes	Yes	Yes	Yes	Yes	Yes	Yes
Controls	Yes	Yes	Yes	Yes	Yes	Yes	Yes
Observations	8830	8890	8950	8920	8860	8870	8980
R-squared	0.474	0.466	0.466	0.464	0.471	0.474	0.477
Number of institutions	883	889	895	892	886	887	898

Robust standard errors in parentheses

***p < 0.01, **p < 0.05, *p < 0.1

**Table 9:** Parallel trends net price, by Cohort

	2012	2013	2014	2015	2016	2017	2018
Treat	0.169**	0.153***	0.150***	0.0326	0.100	0.00649	0.0921***
	(0.0758)	(0.0429)	(0.0395)	(0.0470)	(0.0763)	(0.0951)	(0.0354)
2010	− 0.104***	− 0.1000*	− 0.00811	0.00256	− 0.00289	− 0.00673	0.0233
	(0.0135)	(0.0589)	(0.0368)	(0.0225)	(0.0190)	(0.0103)	(0.0267)
2011	− 0.111**	− 0.132***	0.0306	0.0265	0.0207	0.000815	− 0.0254
	(0.0470)	(0.0459)	(0.0263)	(0.0451)	(0.0400)	(0.0484)	(0.0309)
2012		− 0.119***	0.0316	0.0481*	− 0.0615	− 0.0194	− 0.00309
		(0.0452)	(0.0267)	(0.0291)	(0.0998)	(0.0323)	(0.0342)
2013			0.00729	0.0727*	− 0.138	− 0.000218	− 0.0456
			(0.0322)	(0.0424)	(0.125)	(0.0539)	(0.0362)
2014				0.00439	− 0.178	− 0.0500*	− 0.0644
				(0.0612)	(0.125)	(0.0291)	(0.0490)
2015					− 0.166**	0.0402	− 0.0561
					(0.0836)	(0.0935)	(0.0362)
2016						0.0195	− 0.0665
						(0.0866)	(0.0423)
2017							− 0.0843**
							(0.0407)
Constant	11.24***	11.22***	11.19***	11.27***	11.17***	11.23***	11.25***
	(0.972)	(0.972)	(0.968)	(0.974)	(0.972)	(0.971)	(0.971)
Institution FE	Yes	Yes	Yes	Yes	Yes	Yes	Yes
Year FE	Yes	Yes	Yes	Yes	Yes	Yes	Yes
Controls	Yes	Yes	Yes	Yes	Yes	Yes	Yes
Observations	8830	8890	8950	8920	8860	8870	8980
R-squared	0.061	0.061	0.066	0.060	0.062	0.061	0.061
Number of institutions	883	889	895	892	886	887	898

Robust standard errors in parentheses

***p < 0.01, **p < 0.05, *p < 0.1

**Table 10:** Parallel trends net tuition revenue, by Cohort

	2012	2013	2014	2015	2016	2017	2018
Treat	0.143***	− 0.118	− 0.120*	0.134**	0.113	− 0.0597	0.166
	(0.0254)	(0.121)	(0.0660)	(0.0587)	(0.0733)	(0.0687)	(0.168)
2010	− 0.0270	0.0740*	0.0527	0.00990	0.0460	0.0354*	− 0.0125
	(0.0232)	(0.0398)	(0.0453)	(0.0566)	(0.0655)	(0.0184)	(0.0235)
2011	− 0.00518	0.122***	0.0687	0.0627	0.0434	0.0475*	− 0.0338
	(0.0285)	(0.0438)	(0.0581)	(0.0574)	(0.0624)	(0.0249)	(0.0254)
2012		0.138**	0.128	0.0568	− 0.00843	0.0843**	0.0102
		(0.0603)	(0.0952)	(0.0674)	(0.0285)	(0.0408)	(0.0502)
2013			0.158*	0.000389	− 0.0191	0.0807**	0.00355
			(0.0829)	(0.0544)	(0.0359)	(0.0375)	(0.0456)
2014				− 0.0574	− 0.0121	0.0972**	0.0245
				(0.0815)	(0.0595)	(0.0448)	(0.0579)
2015					− 0.0699	0.0800	− 0.0275
					(0.0666)	(0.0557)	(0.0546)
2016						0.0753	− 0.0445
						(0.0584)	(0.0629)
2017							− 0.0285
							(0.0662)
Constant	10.05***	10.01***	10.05***	10.03***	9.980***	10.05***	10.06***
	(0.635)	(0.636)	(0.636)	(0.643)	(0.608)	(0.636)	(0.630)
Institution FE	Yes	Yes	Yes	Yes	Yes	Yes	Yes
Year FE	Yes	Yes	Yes	Yes	Yes	Yes	Yes
Controls	Yes	Yes	Yes	Yes	Yes	Yes	Yes
Observations	8830	8890	8950	8920	8860	8870	8980
R-squared	0.041	0.043	0.043	0.043	0.041	0.042	0.042
Number of institutions	883	889	895	892	886	887	898

Robust standard errors in parentheses

***p < 0.01, **p < 0.05, *p < 0.1

**Table 11:** Parallel trends non-tuition COA, by Cohort

	2012	2013	2014	2015	2016	2017	2018
Treat	0.0944	0.195**	0.122***	− 0.0239	0.0727	0.0940**	0.106***
	(0.0621)	(0.0840)	(0.0362)	(0.0456)	(0.0619)	(0.0439)	(0.0371)
2010	− 0.0225***	− 0.123	− 0.00666	0.00301	− 0.0153	0.00466	− 0.0189**
	(0.00539)	(0.103)	(0.0143)	(0.00984)	(0.0173)	(0.0161)	(0.00855)
2011	− 0.00158	− 0.122	− 0.0387**	0.00778	− 0.0111	0.00141	− 0.0447**
	(0.0151)	(0.0989)	(0.0163)	(0.0159)	(0.0260)	(0.0231)	(0.0189)
2012		− 0.133**	− 0.0356	0.0231	− 0.0145	− 0.000156	− 0.0470**
		(0.0665)	(0.0240)	(0.0169)	(0.0393)	(0.0223)	(0.0196)
2013			− 0.0585**	0.0796	− 0.0316	− 0.0622	− 0.0605***
			(0.0229)	(0.0501)	(0.0463)	(0.0600)	(0.0232)
2014				0.120**	− 0.0639	− 0.0579	− 0.0682***
				(0.0534)	(0.0721)	(0.0547)	(0.0205)
2015					− 0.0411	− 0.0613	− 0.0702***
					(0.0481)	(0.0395)	(0.0224)
2016						− 0.0682	− 0.0703***
						(0.0429)	(0.0246)
2017							− 0.0661***
							(0.0235)
Constant	9.746***	9.738***	9.715***	9.807***	9.673***	9.739***	9.725***
	(0.358)	(0.364)	(0.352)	(0.360)	(0.358)	(0.356)	(0.359)
Institution FE	Yes	Yes	Yes	Yes	Yes	Yes	Yes
Year FE	Yes	Yes	Yes	Yes	Yes	Yes	Yes
Controls	Yes	Yes	Yes	Yes	Yes	Yes	Yes
Observations	8830	8890	8950	8920	8860	8870	8980
R-squared	0.173	0.172	0.172	0.175	0.173	0.173	0.173
Number of institutions	883	889	895	892	886	887	898

Robust standard errors in parentheses

***p < 0.01, **p < 0.05, *p < 0.1

**Table 12:** Alternative outcome Robustness checks

	Fed appropriations	Fed appropriations	Fed appropriations
Reset	− 99,670	− 111,681	− 544,303
	(119,289)	(116,739)	(458,562)
Year 1		− 51,666	
		(75,865)	
Year 2		− 23,949	
		(63,926)	
Year 3		106,319	
		(78,659)	
Year 4		119,807	
		(90,301)	
Year 5		79,826	
		(103,179)	
Year 6		− 112,878	
		(189,087)	
Reset × size			1.835e+06
			(1.498e+06)
Constant	− 4.754e+06	− 4.742e+06	− 4.679e+06
	(5.473e+06)	(5.469e+06)	(5.455e+06)
R-squared	0.004	0.004	0.004
Number of institutions	944	944	944

Robust standard errors in parentheses

***p < 0.01, **p < 0.05, *p < 0.1

**Table 13:** Callaway and Sant’Anna average treatment effects

	Coef	SE	z	P > z	[95% Conf. interval]	
FTE enrollment	.0665107	.0405944	1.64	0.101	− .0130529	.1460742
Total aid	− .2000465***	.0600651	− 3.33	0.001	− .317772	− .082321
Aid per FTE	− .2665571***	.0466246	− 5.72	0.000	− .3579395	− .1751746
Avg. net price	− .0621349**	.024408	− 2.55	0.011	− .1099737	− .0142961
Per FTE net tuition revenue	− .039814	.0347234	− 1.15	0.252	− .1078707	.0282427
Non-tuition COA	− .0553269***	.0210996	− 2.62	0.009	− .0966813	− .0139725

Robust standard errors in parentheses

***p < 0.01, **p < 0.05, *p < 0.1

**Table 14:** Flexible conditional average treatment effect

	Mean difference					
Outcome	Treatment	Control	DiD	SE	Z	P >|z|
FTE enrollment	0.0265	− 0.0314	0.0578	0.1119	0.5169	0.6078
Total aid	− 0.2035	0.0352	− 0.2387	0.1424	− 1.6763	0.1006
Aid per FTE	− 0.23	0.0666	− 0.2965	0.0889	− 3.3338	0.0017
Avg. net price	− 0.053	− 0.0077	− 0.0453	0.0433	− 1.0457	0.3013
Per FTE net tuition revenue	− 0.0053	− 0.0060	0.0007	0.0448	0.0157	0.9875
Non-tuition COA	0.0086	0.0142	− 0.0057	0.0326	− 0.1738	0.8628

**Table 15:** Two-stage DiD

	FTE enrollment	Total aid	Aid per FTE	Avg. net price	Per FTE net tuition rev	Non-tuition COA
Reset	0.0453	− 0.206***	− 0.251***	− 0.0885***	− 0.0367	− 0.0710***
	(0.0523)	(0.0632)	(0.0432)	(0.0199)	(0.0341)	(0.0216)
Observations	9440	9440	9440	9440	9440	9440

## Footnotes

1 To address this we incorporated advertising expenditures which colleges are required to report on their annual 990 forms they submit to the Internal Revenue Service. We use the 990 data collected and compiled by the Urban Institute (2021). The data are only available from 2011 through 2016. As such, we conducted an additional set of analysis following Callaway and Sant’Anna’s approach to staggered treatment timing using a more limited set of years and treated institutions. Using this limited sample we estimated models with and without the inclusion of advertising dollars and found that this variable had no significant impact on the estimated treatment effects. Given the limited sample included in these analyses, we do not include these findings with the main estimates shown in our findings section. However, these findings assuage concerns that advertising dollars may significantly alter our main estimates. These estimates are available upon request from the corresponding author.

2 Scatter plots comparing reset size to the year-by-year changes of each outcome do not suggest any non-linear relationships. As such, we are comfortable using the linear interpretation to estimate heterogeneous effects.
